# Central nervous system localisation of chronic lymphocytic leukaemia, description of two very distinct cases and a review of the literature

**DOI:** 10.1007/s00277-018-3329-2

**Published:** 2018-04-29

**Authors:** Nyanza K. L. M. Timmers, Josanne S. de Maar, Rob C. M. van Kruijsdijk, Saskia K. Klein

**Affiliations:** 1 Department of Internal Medicine, Academic Medical Centre, Amsterdam, formerly Department of Internal Medicine, Meander Medical Centre, Amersfoort, The Netherlands; 2 Imaging Division, University Medical Centre Utrecht, formerly Department of Internal Medicine, Meander Medical Centre, Amersfoort, The Netherlands; 3 Researchgroup Image Guided Molecular Interventions, University Medical Centre Utrecht, Postbus 85500, Huispostnummer Q 00.3.11, 3508 GA Utrecht, The Netherlands; 4 Department of Internal Medicine, University Medical Centre Utrecht, formerly Department of Internal Medicine, Meander Medical Centre, Amersfoort, The Netherlands; 5 Department of Haematology, Meander Medical Centre, Amersfoort, The Netherlands

**Keywords:** CLL, Chronic lymphocytic leukaemia, Leptomeningeal CLL, Neurological symptoms, Neurological localisation

## Abstract

Central nervous system (CNS) localisation of chronic lymphocytic leukaemia (CLL) can induce various neurological symptoms. Unfamiliarity with this manifestation causes diagnostic delay. We present two cases of leptomeningeal CLL. These cases and our literature review emphasise that CNS localisation of CLL should be considered in patients with any neurological symptom, irrespectively of the stage and systemic activity of CLL.

**Electronic supplementary material:**

The online version of this article (10.1007/s00277-018-3329-2) contains supplementary material, which is available to authorized users.

## Introduction

Chronic lymphocytic leukaemia (CLL) is a lymphoproliferative disorder of monoclonal B cells and is classically characterised by lymphocytosis, lymphadenopathy and lymphoid infiltration of the bone marrow [1]. It is the most common leukaemia among adults in Western countries, with an incidence of 808 patients per 100,000 in 2015 [2]. Most patients are diagnosed incidentally due to routine blood investigation, while others present with non-specific symptoms, lymphadenopathy, infections, or B-symptoms. The presence of lymphocyte infiltration in extramedullary localisations, such of as the central nervous system (CNS), causing neurological symptoms is rare. So far, only 170 individual cases of CNS involvement of CLL have been described, although autopsy studies showed CNS localisation in at least 20% of CLL patients [3], emphasising the necessity of increasing awareness. A recent cohort study of 4174 patients with chronic lymphocytic leukaemia showed that 0.4% had symptomatic central nervous system involvement [4].

In order to stress the heterogeneity of the disorder, the importance of early recognition and the diagnostic challenges, we present two very distinct cases of CNS localisation of CLL and we performed a review of the literature.

## Case descriptions

### Case 1

An 81-year-old woman diagnosed 20 months earlier with CLL (Rai 0, Binet A), who had been followed by a wait-and-see policy, was referred to our hospital due to progressive diplopia since 2 weeks. Initial evaluation by the ophthalmologist had not clarified the cause of her symptoms. On admission, the patient had a paralysis of the left oculomotor nerve and left hemianopsia, without any other signs or symptoms. The peripheral blood counts showed a haemoglobin of 7.2 mmol/L, with a leukocyte count of 33.4 × 10^9^/L and platelets of 163 × 10^9^/L. A few months earlier, she had had a leukocyte count of 39.9 × 10^9^/L with a lymphocyte count of 35.6 × 10^9^/L. On the cerebral computed tomography (CT) and magnetic resonance imaging (MRI), no relevant abnormalities were observed. Lumbar puncture (LP) results showed an elevated cerebrospinal fluid (CSF) protein (907 mg/L) and elevated leukocytes (67.0 × 10^6^/L). Immunophenotyping showed a monoclonal B cell population in 5% of the leukocytes. As the cerebrospinal fluid sample did not contain any erythrocytes, contamination with peripheral blood was not considered likely. The diagnosis of leptomeningeal CLL was established (Table 1).

**Table 1:** Immunophenotyping and cytogenetic analysis

	Case 1	Case 2
Immunophenotyping at time of initial CLL diagnosis (peripheral blood)	Weak SmIgL+/weak SmIgD+/CD19+/weak CD20+/CD45+/CD10−/weakCD22+/CD5+/CD23+/weak FMC7+/CD103−/CD11c−/weak CD25+/CD24+/CD52+/weak CD38+/weak CD79b+ Kappa/lambda ratio < 0.1	CD19+/weak SmIgKappa+/very weak SmIgM+/SmIgD+/CD20+/CD22+/CD23+/CD5+/FMC7−/CD52+/partially weak CD38+ Kappa/lambda ratio 1.70
Cytogenetics at time of initial CLL diagnosis (bone marrow)	Unknown	46XY deletion 6q−, deletion 11q− and translocation (7;8). Breakpoint distally from 11q22ATM
Immunophenotyping at time of CNS localisation (cerebrospinal fluid)	Weak SmIgL+/CD19+/weak CD20+/weak CD5+ Kappa/lambda ratio 0.01	CD19+/light chain negative/weak CD22+/weak CD5+/CD23+/CD24+/CD43+/weak CD81+ Kappa/lambda ratio 1.23
Immunophenotyping at time of CNS localisation (bone marrow)	Weak SmIgL+/Weak SmIgD+/CD19+/weak CD20+/weak CD45+/CD10−/weak CD22+/weak CD5+/CD23+/FMC7−/CD103−/CD11c−/weak CD25+/CD24+/weak CD38+/CD79b−	Unknown
Cytogenetics at time of CNS localisation (bone marrow)	17p deletion (17p13.1) without additional abnormalities	Unknown

*CLL* chronic lymphocytic leukaemia, *CNS* central nervous system

Intrathecal methotrexate (IT-MTX) was started, and the patient was discharged. However, after three treatment courses, she was readmitted with malaise and pancytopenia, ascribed to CLL progression. Treatment with rituximab and chlorambucil (R-chlorambucil) was commenced. A rapid progression of her prior mild cognitive impairment was objectified. MRI and LP showed no signs of CLL progression or encephalitis. Hence, the decline was accounted to the IT-MTX [5], although a causal effect of leptomeningeal CLL cannot be ruled out. Despite of her weak condition, treatment with IT-MTX and R-chlorambucil was completed. Finally, her general condition stabilised and her vision improved considerably.

### Case 2

A 77-year-old man who had been diagnosed 9 years earlier with CLL (Rai 2, Binet B) and was followed by watchful waiting policy after initial treatment with fludarabine and cyclophosphamide, was referred to our emergency department due to apathy. Since months, he had been easily fatigued. A few days before presentation, he had developed urinary incontinence and a non-productive cough. On examination, he was conscious but barely reacted to speech and was tachypneic. Laboratory results showed a haemoglobin of 8.5 mmol/L, a leukocyte count of 48.0 × 10^9^/L, of which 44.5 × 10^9^/L lymphocytes, and 104 × 10^9^/L thrombocytes. C-reactive protein was slightly elevated to 34 mg/L.

For a suspected pneumonia causing a hypo-active delirium, treatment with meropenem was started. The day after admission, the patient was transferred to the intensive care unit due to a declining consciousness and impending respiratory failure. Neuroimaging showed a communicating hydrocephalus without leptomeningeal enhancement, signs of a herpes encephalitis or sinus thrombosis. Initial LP did not show an elevated pressure, and there were no cellular abnormalities by routine analysis, nor bacterial or viral pathogens.

After initial slight improvement, the apathy worsened. A second LP, repeated for immunological evaluation, showed an abnormal B cell population in 2% of the leukocytes confirming leptomeningeal CLL (Table 1). No erythrocytes, granulocytes or monocytes were visible in the cerebrospinal fluid, making contamination with peripheral blood very unlikely.

Considering the patient’s poor clinical condition, no viable therapeutic options were available. The patient died 4 days after the diagnosis of leptomeningeal CLL.

## Methods and materials

We conducted a review of available literature on CNS localisation of CLL using PubMed and EMBASE (supplement 1 and 2). We excluded Richter’s transformation, mature T cell neoplasms and cases with a second haematological malignancy as primary cause of the CNS symptoms.

## Results and discussion

We presented two cases of CLL with CNS localisation with leptomeningeal infiltration. Notable is the heterogeneity in symptoms, severity, clinical course and outcome of our two cases. This characterises CNS involvement but simultaneously poses challenges in the diagnosis and provides a risk for doctors’ delay. As illustrated by our cases and supported by our literature search, CNS localisation is difficult to recognise due to its unfamiliarity. This highlights the need for more awareness of this manifestation.

### General findings literature search

Previous authors have conducted literature reviews on the subject, some only concerning specific subcategories [6–16]. In contrast to the most recent review by de Souza et al. [14], describing 92 patients with CNS involvement of CLL, we did not focus specifically on immunophenotyping, but on the clinical presentation and complete diagnostic process [14].

Our literature search showed 170 unique cases of CNS localisation in CLL, reported between 1973 and 2017 (supplement 3). A summary of our findings is displayed in Table 2. The mean age of reported patients was 64.5 years, 59.7% was male. The time between initial diagnosis of CLL and the development of neurologic symptoms varied substantially. The described average time in literature was 32.3 months, with a range of 0–144 months. In comparison, our first case developed neurological symptoms after 20 months, while the gap in our second case was 9 years. In 27.9% of patients, neurological symptoms were present at diagnosis of CLL. Counterintuitively, previous studies did not show a clear correlation between the occurrence of CNS localisation and CLL Rai stage at time of onset [12, 13]. This highlights the necessity of incorporating CNS localisation in the differential diagnosis in any patient with CLL and neurological symptoms, irrespectively of time of diagnosis or disease activity.

**Table 2:** Overview of literature

Number of articles		89
Number of individual cases		170
Sex, *n* (%)	Male	74 (59.7%)
	Female	50 (40.3%)
	Not reported	46
Mean age		64.5 years
Mean time interval from diagnosis of CLL to neurological symptoms		32.3 months
Most common neurological symptoms^a^, allocated to CNS localisation of CLL, *n* (% of 125 patients with known symptoms)	Symptoms unknown	45
	No symptoms	2 (1.6%)
	Symptoms attributed to cranial nerve palsy (any)	64 (51.2%)
	- Diplopia (N. III/IV/VI)	22 (17.6%)
	- Impaired vision (N. II)	22 (17.6%)
	- Facial palsy (N. VII)	11 (8.8%)
	- Impaired hearing	7 (5.6%)
	- Impaired visual field	7 (5.6%)
	- Dysarthria (N. V/VII/IX/X/XII)	6 (4.8%)
	- Other	11 (8.8%)
	Headache	30 (24.0%)
	Altered mental state	19 (15.2%)
	Cognitive decline	16 (12.8%)
	Limb paralysis/paresis	15 (12.0%)
	Impaired coordination of extremities	15 (12.0%)
	Dizziness/vertigo	9 (7.2%)
	Radiculopathy	8 (6.4%)
	Other symptoms	81 (68.1.%)
Localisation of CLL, *n* (% of 120 patients with known localisation)	Localisation unknown	50
	Leptomeningeal	66 (55.0%)
	Cerebral hemisphere	20 (16.7%)
	Multiple sites	11 (9.2%)
	Spinal cord or roots	9 (7.5%)
	1 specific cranial nerve	7 (5.8%)
	Hypophysis/pituitary gland	3 (2.5%)
	Cerebellum	2 (1.7%)
	Dural	1 (0.8%)
	Brain stem	1 (0.8%)
Method of diagnosis^b^, *n* (% of 152 patients with known method of diagnosis)	Method of diagnosis unknown	18
	CSF	118 (77. 6%)
	MRI brain or spinal cord	52 (34.2%)
	Biopsy	30 (19.7%)
	Obduction	13 (8.6%)
	CT brain	11 (7.2%)

*CLL* chronic lymphocytic leukaemia, *CNS* central nervous system, *CSF* cerebrospinal fluid, *MRI* magnetic resonance imaging, *CT* computed tomography

^a^Multiple symptoms may be present in one patient

^b^Multiple methods may have been used in one patient

### Clinical symptoms and relation to presented cases

The heterogeneity of clinical symptoms at presentation makes recognising CNS localisation of CLL challenging. More than half of the patients (51.2%) described in the literature presented with symptoms accounted to one or more cranial nerve palsies. Diplopia and impaired vision were the most common, both present in 17.6% of the cases (Table 2). In our first case, the symptom at presentation was diplopia. As our patient did not exhibit any systemic CLL symptoms or signs of disease progression, our patient had first been referred to the optician and ophthalmologist who referred the patient to the neurologist. The differential diagnosis consisted of an intracranial tumour, stroke, aneurysm or infection. Furthermore, as there was no progression of leucocytosis, CLL disease progression was not suspected at that point. Hence, an insidious presentation of CNS involvement of CLL, without systemic symptoms, is not unusual. Early recognition of the possible relation between CLL and cranial nerve palsies can prevent unnecessary diagnostic delay.

Not all patients with leptomeningeal CLL present with specific symptoms of the cranial nerves. Of the patients described in the literature, 15.2% presented with an altered mental state and 12.8% had a cognitive decline (Table 2). Non-specific neurologic symptoms, as seen in our second case, are common in CNS involvement of CLL. Our second patient presented with an altered mental state, which in retrospect had been slowly progressive over a few weeks with an acute decline on the day of admission. He was thought to have a bacterial pneumonia with a hypo-active delirium. As in our first case, cerebral imaging did not contribute to the diagnosis. Only when the apathy remained despite treatment, other causes were considered, including viral encephalitis. When further evaluation did not confirm an alternative diagnosis, leptomeningeal CLL was suspected. The timing of the change in mental state in our second case was indicative of the underlying cause; although the history was too long for a delirium, it progressed too fast to be attributed to cognitive decline as generally seen in dementia.

### Localisation and diagnostic procedure

Both patients presented with leptomeningeal CLL, which is the most common CNS localisation described in the literature (55.0%). However, this may be biased, as neuroimaging is not always performed, possibly missing localised brain or spinal cord involvement. Contrast-enhanced MRI is the preferred imaging modality. Although no golden standard testing is available for CNS localisation of CLL, CSF examination plays a key role in the diagnosis. In 43.4% of the cases described in the literature, the diagnosis was purely based on CSF examination. However, in our second case and in at least 3.9% of the cases described in the literature, cytological examination only of the CSF was non-diagnostic. Therefore, whenever CNS involvement is suspected, CSF cytology should be combined with immunophenotyping. Of note, immunophenotyping might be false positive if the cerebrospinal fluid sample includes even a small amount of peripheral blood. Thus, the diagnosis of CNS involvement cannot be confirmed if erythrocytes are present in the cerebrospinal fluid sample.

### Treatment

Current guidelines do not provide recommendations on the treatment of patients with CNS involvement of CLL [17]. Several different treatment regimes have been described with varying degrees of success. In a retrospective cohort of 30 patients, systemic as well as intrathecal chemotherapy was applied with and without rituximab. Furthermore, some patients were treated with rituximab monotherapy, localised radiotherapy or the BTK inhibitor ibrutinib [18]. Recently, ibrutinib, a Bruton’s tyrosine kinase inhibitor that penetrates the blood-brain barrier, has shown positive results in patients with CNS localisation of CLL [19] and other lymphoproliferative diseases [20, 21]. Although further research is necessary to confirm these results, it seems a promising option for patients with CNS localisation of CLL [18].

We could not find any reports on penetration of the blood-brain barrier in CNS tumours by PI3K inhibitors (idelalisib) or BCL2 antagonists (venetoclax). Given the apparent frailty and absence of systemic symptoms in the patient described in case 1, intrathecal administration of MTX was considered preferential over systemic therapy. However, when she developed systemic symptoms, treatment with R-chlorambucil was commenced according to international guidelines for less fit patients [17]. At that time, BTK inhibitors were not available for regular use. In case 2, the detrimental clinical condition of the patient did not allow for initiation of treatment. Naturally, tailored treatment remains crucial, taking into account the presence of systemic symptoms, patient fitness and comorbidities, and previously administered therapy. New, emerging therapeutic options should be followed closely, considering that small molecules might overcome limitations (such as difficulty in passing the blood-brain barrier) of former treatment options.

## Conclusion

CNS localisation of CLL should be considered in patients with chronic lymphocytic leukaemia presenting with any neurological symptom, irrespectively of the stage and systemic activity of the disease. Immunophenotyping of the cerebrospinal fluid should always be part of the diagnostic workup, as neuroimaging and CSF cytology can be normal.

## Electronic supplementary material

ESM 1 (PDF 223 kb)

## References

[1] Chiorazzi N, Rai KR, Ferrarini M (2005). Chronic lymphocytic leukemia. N Engl J Med.

[2] Integraal kankercentrum Nederland. Dutch cancer figures—incidence hematology / B-CLL / small cell B-cell lymphoma. Available at: http://www.cijfersoverkankernl/selecties/Dataset_2/img587c91a64ce66. 2016(Updated 04–02-2016, the rates for 2015 are based on estimations.)

[3] Barcos M, Lane W, Gomez GA, Han T, Freeman A, Preisler H, Henderson E (1987). An autopsy study of 1206 acute and chronic leukemias (1958 to 1982). Cancer.

[4] Strati P, Uhm JH, Kaufmann TJ, Nabhan C, Parikh SA, Hanson CA, Chaffee KG, Call TG, Shanafelt TD (2016). Prevalence and characteristics of central nervous system involvement by chronic lymphocytic leukemia. Haematologica.

[5] Finkelstein Y, Zevin S, Raikhlin-Eisenkraft B, Bentur Y (2005). Intrathecal methotrexate neurotoxicity: clinical correlates and antidotal treatment. Environ Toxicol Pharmacol.

[6] Cramer SC, Glaspy JA, Efird JT, Louis DN (1996). Chronic lymphocytic leukemia and the central nervous system: a clinical and pathological study. Neurology.

[7] Miller K, Budke H, Orazi A (1997). Leukemic meningitis complicating early stage chronic lymphocytic leukemia. Arch Pathol Lab Med.

[8] Morrison C, Shah S, Flinn IW (1998). Leptomeningeal involvement in chronic lymphocytic leukemia. Cancer Pract.

[9] Brick WG, Majmundar M, Hendricks LK, Kallab AM, Burgess RE, Jillella AP (2002). Leukemic leptomeningeal involvement in stage 0 and stage 1 chronic lymphocytic leukemia. Leuk Lymphoma.

[10] Giordano A, Perrone T, Guarini A, Ciappetta P, Rubini G, Ricco R, Palma M, Specchia G, Liso V (2007). Primary intracranial dural B cell small lymphocytic lymphoma. Leuk Lymphoma.

[11] Lange CP, Brouwer RE, Brooimans R, Vecht Ch J (2007). Leptomeningeal disease in chronic lymphocytic leukemia. Clin Neurol Neurosurg.

[12] Moazzam AA, Drappatz J, Kim RY, Kesari S (2012). Chronic lymphocytic leukemia with central nervous system involvement: report of two cases with a comprehensive literature review. J Neuro-Oncol.

[13] Lopes da Silva R (2012). Spectrum of neurologic complications in chronic lymphocytic leukemia. Clin Lymphoma Myeloma Leuk.

[14] de Souza SL, Santiago F, de Moura Ribeiro-Carvalho M, Arnóbio A, Rebeiro Soares A, Ornellas MH (2014). Leptomeningeal involvement in B-cell chronic lymphocytic leukemia: a case report and review of the literature. BMC Res Notes.

[15] Zhu J, Wu Z, Fan L, Li J (2014). Chronic lymphocytic leukemia with central nervous system invasion: one case report and literature review. Zhonghua xue ye xue za zhi.

[16] Khan K, Malik AI, Almarzouqi SJ, Morgan ML, Yalamanchili S, Chevez- Barrios P, Lee AG (2016). Optic neuropathy due to chronic lymphocytic leukemia proven with optic nerve sheath biopsy. J Neuroophthalmol.

[17] Eichhorst B, Robak T, Montserrat E, Ghia P, Hillmen P, Hallek M, Buske C, Committee EG (2015). Chronic lymphocytic leukaemia: ESMO clinical practice guidelines for diagnosis, treatment and follow-up. Ann Oncol.

[18] Wanquet A, Birsen R, Bonnet C, Boubaya M, Choquet S, Dupuis J, Lepretre S, Re D, Fahri J, Michallet AS, Ysebaert L, Lemal R, Lamy T, Delarue R, Troussard X, Cymbalista F, Levy V, Dietrich PY, Leblond V, Aurran-Schleinitz T (2017). Management of central nervous system involvement in chronic lymphocytic leukaemia: a retrospective cohort of 30 patients. Br J Haematol.

[19] Wanquet A, Birsen R, Lemal R, Hunault M, Leblond V, Aurran-Schleinitz T (2016). Ibrutinib responsive central nervous system involvement in chronic lymphocytic leukemia. Blood.

[20] Bernard S, Goldwirt L, Amorim S, Brice P, Brière J, de Kerviler E, Mourah S, Sauvageon H, Thieblemont C (2015). Activity of ibrutinib in mantle cell lymphoma patients with central nervous system relapse. Blood.

[21] Cabannes-Hamy A, Lemal R, Goldwirt L, Poulain S, Amorim S, Pérignon R, Berger J, Brice P, De Kerviler E, Bay J-O, Sauvageon H, Beldjord K, Mourah S, Tournilhac O, Thieblemont C (2016). Efficacy of ibrutinib in the treatment of Bing–Neel syndrome. Am J Hematol.

