# Supplementary material for: Central nervous system localisation of chronic lymphocytic leukaemia, description of two very distinct cases and a review of the literature
Source: Ann Hematol. 2018 Apr 29;97(9):1627–32. doi: 10.1007/s00277-018-3329-2 (PMC6097746; doi:10.1007/s00277-018-3329-2)
Supplement: Supplementary file 1 — (PDF 223 kb) [file 277_2018_3329_MOESM1_ESM.pdf]

1 SUPPLEMENT 1 SEARCH TERMS LITERATURE REVIEW

**Search strategy in PubMed and EMBASE**		
**Database**	**Search strategy**	**Hits**
**PubMed**	**# 1 Chronic Lymphocytic leukemia**<br>(((((B-CLL[Title/Abstract]) OR chronic lymphocytic leukemia[MeSH Terms]) OR chronic lymphocytic leukemias[MeSH Terms]) OR CLL[Title/Abstract])) OR (((((chronic[Title/Abstract] OR B-chronic[Title/Abstract]))) AND ((lymphatic[Title/Abstract] OR lymphocyte[Title/Abstract] OR lymphoid[Title/Abstract] OR lymphocytic[Title/Abstract] OR “B-lymphatic”[Title/Abstract] OR “B-lymphocytic”[Title/Abstract] OR “B-lymphocyte”[Title/Abstract] OR “B-lymphoid”[Title/Abstract] OR “B-cell”[Title/Abstract] OR “B cell”[Title/Abstract]))) AND ((leukemia*[Title/Abstract] OR leucemia*[Title/Abstract] OR leukaemia*[Title/Abstract] OR leucaemia*[Title/Abstract])))	27776
	**#2 Central nervous system localisation / symptoms**<br>(((((neurologic[Title/Abstract] OR leptomeningeal[Title/Abstract] OR nerv*[Title/Abstract] OR mening*[Title/Abstract] OR encephal*[Title/Abstract])) AND (complication*[Title/Abstract] OR symptom*[Title/Abstract] OR disease*[Title/Abstract] OR feature*[Title/Abstract] OR syndrom*[Title/Abstract] OR manifestation*[Title/Abstract] OR involvement*[Title/Abstract]))) OR ((“cerebrospinal fluid”[Title/Abstract] OR “spinal fluid”[Title/Abstract] OR “spinal cord”[Title/Abstract] OR “spinal cords”[Title/Abstract] OR hypophysis[Title/Abstract] OR hypothalam*[Title/Abstract] OR pituitary[Title/Abstract] OR neuropath*[Title/Abstract] OR leukoencephalopath*[Title/Abstract]))) OR ((central nervous system[MeSH Terms]) OR (“nervous system”[Title/Abstract] OR cns[Title/Abstract] OR “nerve system”[Title/Abstract] OR “nervous tissue”[Title/Abstract] OR “systema nervosum”[Title/Abstract]))	1821812

	**#3: #1 AND #2**<br>(((((((neurologic[Title/Abstract] OR leptomeningeal[Title/Abstract] OR nerv*[Title/Abstract] OR mening*[Title/Abstract] OR encephal*[Title/Abstract])) AND (complication*[Title/Abstract] OR symptom*[Title/Abstract] OR disease*[Title/Abstract] OR feature*[Title/Abstract] OR syndrom*[Title/Abstract] OR manifestation*[Title/Abstract] OR involvement*[Title/Abstract]))) OR ((“cerebrospinal fluid”[Title/Abstract] OR “spinal fluid”[Title/Abstract] OR “spinal cord”[Title/Abstract] OR “spinal cords”[Title/Abstract] OR hypophysis[Title/Abstract] OR hypothalam*[Title/Abstract] OR pituitary[Title/Abstract] OR neuropath*[Title/Abstract] OR leukoencephalopath*[Title/Abstract]))) OR ((central nervous system[MeSH Terms]) OR (“nervous system”[Title/Abstract] OR cns[Title/Abstract] OR “nerve system”[Title/Abstract] OR “nervous tissue”[Title/Abstract] OR “systema nervosum”[Title/Abstract])))) AND ((((((B-CLL[Title/Abstract]) OR chronic lymphocytic leukemia[MeSH Terms]) OR chronic lymphocytic leukemias[MeSH Terms]) OR CLL[Title/Abstract])) OR (((((chronic[Title/Abstract] OR B-chronic[Title/Abstract]))) AND ((lymphatic[Title/Abstract] OR lymphocyte[Title/Abstract] OR lymphoid[Title/Abstract] OR lymphocytic[Title/Abstract] OR “B-lymphatic”[Title/Abstract] OR “B-lymphocytic”[Title/Abstract] OR “B-lymphocyte”[Title/Abstract] OR “B-lymphoid”[Title/Abstract] OR “B-cell”[Title/Abstract] OR “B cell”[Title/Abstract]))) AND ((leukemia*[Title/Abstract] OR leucemia*[Title/Abstract] OR leukaemia*[Title/Abstract] OR leucaemia*[Title/Abstract]))))	719
**EMBASE**	**#4 Chronic Lymphocytic leukemia**<br>'chronic lymphatic leukemia'/exp OR 'chronic lymphatic leukemia' OR cll:ab,ti OR 'b cll':ab,ti OR 't cll':ab,ti OR (chronic:ab,ti OR 'b chronic':ab,ti OR 't chronic':ab,ti AND (lymphatic:ab,ti OR lymphocyte:ab,ti OR lymphoid:ab,ti OR lymphocytic:ab,ti OR 'b-lymphatic':ab,ti OR 'b-lymphocytic':ab,ti OR 'b-	51067

	lymphocyte':ab,ti OR 'b-lymphoid':ab,ti OR 'b-cell':ab,ti OR 'b cell':ab,ti) AND (leukemia*:ab,ti OR leucemia*:ab,ti OR leukaemia*:ab,ti OR leucaemia*:ab,ti))	
	**#5 Central nervous system localisation / symptoms** 'central nervous system'/exp OR 'nervous system':ab,ti OR cns:ab,ti OR 'nerve system':ab,ti OR 'nervous tissue':ab,ti OR 'systema nervosum':ab,ti OR mening*:ab,ti OR encephal*:ab,ti OR 'cerebrospinal fluid':ab,ti OR 'spinal fluid':ab,ti OR 'spinal cord':ab,ti OR 'spinal cords':ab,ti OR hypophysis:ab,ti OR hypothalam*:ab,ti OR pituitary:ab,ti OR nerv*:ab,ti OR neuropath*:ab,ti OR leukoencephalopath*:ab,ti OR (neurologic:ab,ti OR leptomeningeal:ab,ti AND (complication*:ab,ti OR symptom*:ab,ti OR disease*:ab,ti OR feature*:ab,ti OR syndrom*:ab,ti OR manifestation*:ab,ti OR involvement*:ab,ti))	2835586
	**#6: #4 AND #5** 'chronic lymphatic leukemia'/exp OR 'chronic lymphatic leukemia' OR cll:ab,ti OR 'b cll':ab,ti OR 't cll':ab,ti OR (chronic:ab,ti OR 'b chronic':ab,ti OR 't chronic':ab,ti AND (lymphatic:ab,ti OR lymphocyte:ab,ti OR lymphoid:ab,ti OR lymphocytic:ab,ti OR 'b-lymphatic':ab,ti OR 'b-lymphocytic':ab,ti OR 'b-lymphocyte':ab,ti OR 'b-lymphoid':ab,ti OR 'b-cell':ab,ti OR 'b cell':ab,ti) AND (leukemia*:ab,ti OR leucemia*:ab,ti OR leukaemia*:ab,ti OR leucaemia*:ab,ti)) AND ('central nervous system'/exp OR 'nervous system':ab,ti OR cns:ab,ti OR 'nerve system':ab,ti OR 'nervous tissue':ab,ti OR 'systema nervosum':ab,ti OR mening*:ab,ti OR encephal*:ab,ti OR 'cerebrospinal fluid':ab,ti OR 'spinal fluid':ab,ti OR 'spinal cord':ab,ti OR 'spinal cords':ab,ti OR hypophysis:ab,ti OR hypothalam*:ab,ti OR pituitary:ab,ti OR nerv*:ab,ti OR neuropath*:ab,ti OR leukoencephalopath*:ab,ti OR (neurologic:ab,ti OR leptomeningeal:ab,ti AND (complication*:ab,ti OR symptom*:ab,ti OR disease*:ab,ti OR feature*:ab,ti OR syndrom*:ab,ti OR manifestation*:ab,ti OR involvement*:ab,ti)))	1947
	**#7: #6 AND [embase]/lim NOT [medline]/lim**	**984**

'chronic lymphatic leukemia'/exp OR 'chronic lymphatic leukemia' OR cll:ab,ti OR 'b cll':ab,ti OR (chronic:ab,ti OR 'b chronic':ab,ti AND (lymphatic:ab,ti OR lymphocyte:ab,ti OR lymphoid:ab,ti OR lymphocytic:ab,ti OR 'b-lymphatic':ab,ti OR 'b-lymphocytic':ab,ti OR 'b-lymphocyte':ab,ti OR 'b-lymphoid':ab,ti OR 'b-cell':ab,ti OR 'b cell':ab,ti) AND (leukemia*:ab,ti OR leucemia*:ab,ti OR leukaemia*:ab,ti OR leucaemia*:ab,ti)) AND ('central nervous system'/exp OR 'nervous system':ab,ti OR cns:ab,ti OR 'nerve system':ab,ti OR 'nervous tissue':ab,ti OR 'systema nervosum':ab,ti OR 'cerebrospinal fluid':ab,ti OR 'spinal fluid':ab,ti OR 'spinal cord':ab,ti OR 'spinal cords':ab,ti OR hypophysis:ab,ti OR hypothalam*:ab,ti OR pituitary:ab,ti OR neuropath*:ab,ti OR leukoencephalopath*:ab,ti OR (neurologic:ab,ti OR leptomeningeal:ab,ti OR nerv*:ab,ti OR mening*:ab,ti OR encephal*:ab,ti AND (complication*:ab,ti OR symptom*:ab,ti OR disease*:ab,ti OR feature*:ab,ti OR syndrom*:ab,ti OR manifestation*:ab,ti OR involvement*:ab,ti))) AND [embase]/lim NOT [medline]/lim	
**Total**	**1667**
Search conducted on August 30th 2017	

SUPPLEMENT 2 FLOWCHART LITERATURE REVIEW

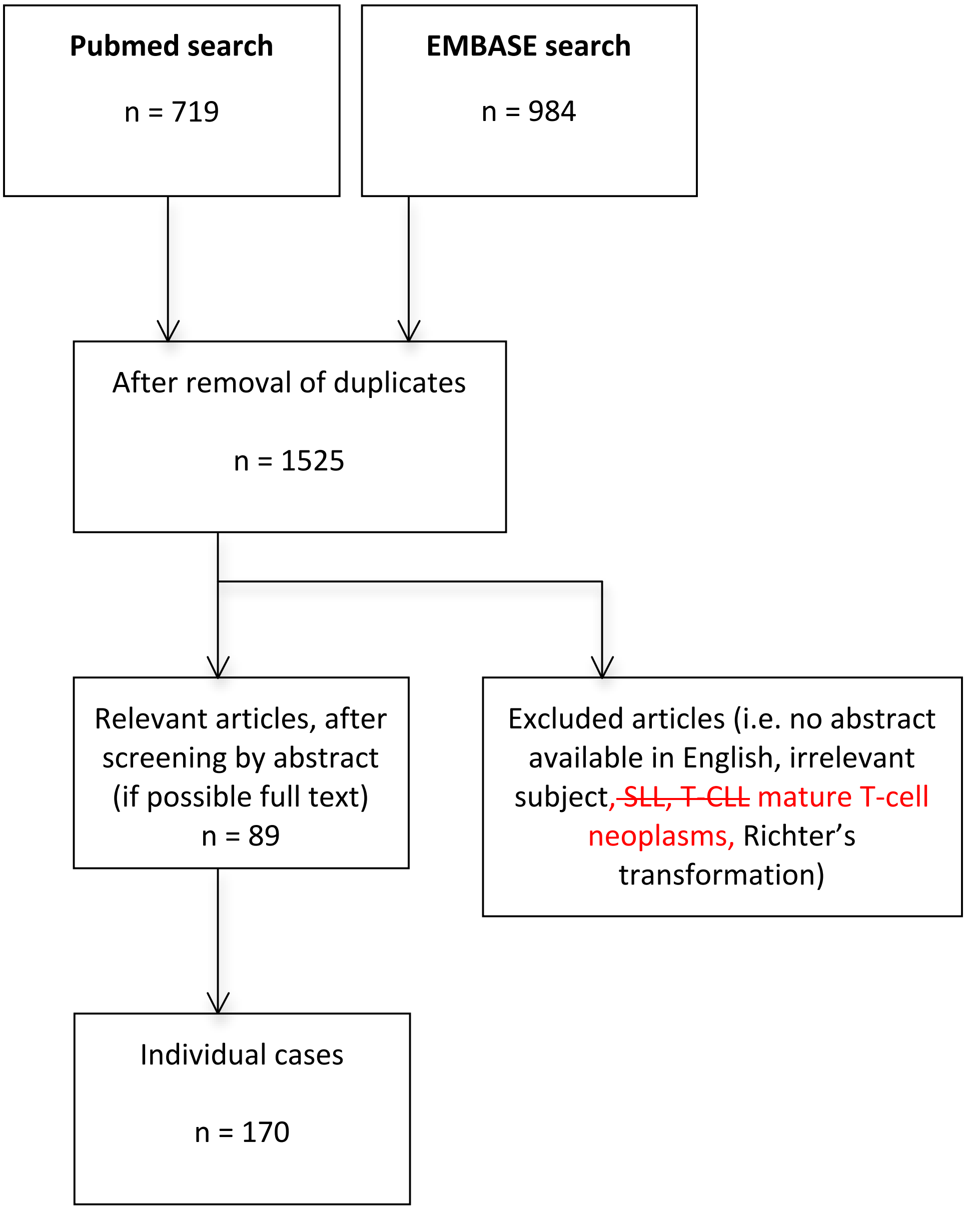

SUPPLEMENT 3 REFERENCE LIST OF THE LITERATURE REVIEW

1. Afifi AM. Unusual ocular manifestations in neoplasia of the lymphoreticular system. Ain Shams Medical Journal. 1973;24(5):341-5.

2. Akintola-Ogunremi O, Whitney C, Mathur SC, Finch CN. Chronic lymphocytic leukemia presenting with symptomatic central nervous system involvement. Ann Hematol. 2002;81(7):402-4.

3. Benjamini O, Jain P, Schlette E, Sciffman JS, Estrov Z, Keating M. Chronic lymphocytic leukemia with central nervous system involvement: a high-risk disease? Clin Lymphoma Myeloma Leuk. 2013;13(3):338-41.

4. Beris P, Miescher PA, Wildi E. Progressive encephalopathy in three cases of chronic lymphatic leukemia. Schweizerische Medizinische Wochenschrift 1980;110(2):437-46.

5. Boogerd W, Vroom TM. Meningeal involvement as the initial symptom of B cell chronic lymphocytic leukemia. European Neurology. 1986;25(6):461-4.

6. Bower JH, Hammack JE, McDonnell SK, Tefferi A. The neurologic complications of B-cell chronic lymphocytic leukemia. Neurology. 1997;48:407-12.

7. Brick WG, Majmundar M, Hendricks LK, Kallab AM, Burgess RE, Jillella AP. Leukemic Leptomeningeal Involvement in Stage 0 and Stage 1 Chronic Lymphocytic Leukemia. Leukemia & Lymphoma. 2002;43:199-201.

8. Brucker P, Lederlin P, Floquet J. Meningeal lymphocytosis in chronic lymphoid leukemia with hypercytosis. Annales Medicales de Nancy. 1977;16(1):51-4.

9. Cash J, Fehir KM, Pollack MS. Meningeal Involvement in Early Stage Chronic Lymphocytic Leukemia. Cancer. 1987;59:798-800.

10. Chantepie SP, Cornet E. Leptomeningeal involvement in CLL. Blood Res. 2014;49(1):5.

11. Cohen JB, Cavaliere R, Byrd JC, Andritsos LA. Hearing Loss due to Infiltration of the Tympanic Membrane by Chronic Lymphocytic Leukemia. Case Rep Hematol. 2012;2012:589718.

12. Conesa V, Mompel A, Ruiz J, et al. Fulminant Brain Lymphoid Infiltration in a Patient With Chronic Lymphocytic Leukemia. American Journal of Hematology. 1999;60:167-72.

13. Cramer SC, Glaspy JA, Efird JT, Louis DN. Chronic lymphocytic leukemia and the central nervous system: A clinical and pathological study. Neurology. 1996;46:19-25.

14. Currie JN, Lessell S, Lessell IM, Weiss JS, Albert DM, Benson EM. Optic Neuropathy in Chronic Lymphocytic Leukemia. Arch Ophthalmol. 1988;106:654-60.

15. de Souza SL, Santiago F, de Moura Ribeiro-Carvalho M, Arnóbio A, Rebeiro Soares A, Ornellas MH. Leptomeningeal involvement in B-cell chronic lymphocytic leukemia: a case report and review of the literature. BMC Research Notes. 2014;7.

16. Denier C, Tertian G, Ribrag V, et al. Multifocal deficits due to leukemic meningoradiculitis in chronic lymphocytic leukemia. Journal of the Neurological Sciences. 2009;277(1-2):130-2.

17. Diwan RV, Diwan VG, Bellon EM. Brain involvement in Chronic Lymphocytic Leukemia. Journal of Computer Assisted Tomography. 1982;6(4):82-814.

18. Elliott MA, Letendre L, Li C-Y, Hoyer JD, Hammack JE. Chronic lymphocytic leukaemia with symptomatic diffuse central nervous system infiltration responding to therapy with systemic fludarabine. British Journal of Haematology. 1999;104:689-94.

19. Fain JS, Naeim F, Becker DP, et al. Chronic lymphocytic leukemia presenting as a pituitary mass lesion. Canadian Journal of Neurological Sciences. 1992;19(2):239-42.

20. Faivre G, Singer S, DeAngelis L, Noy A, Omuro A. Rituximab and bendamustine for CNS involvement by chronic lymphocytic leukemia. Neurology. 2014;82(10 Suppl. 1).

21. Garicochea B, Cliquet MG, Melo N, Del Giglio A, Dorlhiac-Llacer PE, Chamone DAF. Leptomeningeal involvement in chronic lymphocytic leukemia identified by polymerase chain reaction in stored slides: A case report. Modern Pathology 1997;10(5):500-3.

22. Garofalo M, Murali R, Halperin I, Magardician K, Moussouris HF, Masdeu JC. Chronic Lymphocytic Leukemia With Hypothalamic Invasion. Cancer. 1989;64:1714-6.

23. Gétaz EP, Miller GJ. Spinal cord involvement in chronic lymphocytic leukemia. Cancer. 1979;43:1858-61.

24. Giordano A, Perrone T, Guarini A, et al. Primary intracranial dural B cell small lymphocytic lymphoma. Leuk Lymphoma. 2007;48(7):1437-43.

25. Gobbi M, Tazzari PL, Raspadori D. Meningeal leukemia complicating prolymphocytoid transformation of B-chronic lymphocytic leukemia. Acta Haematologica. 1985;74(4):205-7.

26. Gunther JR, Rahman AR, Dong W, et al. Craniospinal irradiation prior to stem cell transplant for hematologic malignancies with CNS involvement: Effectiveness and toxicity after photon or proton treatment. Pract Radiat Oncol. 2017;S1879-8500(17).

27. Hanse MC, Van't Veer MB, van Lom K, van den Bent MJ. Incidence of central nervous system involvement in chronic lymphocytic leukemia and outcome to treatment. J Neurol. 2008;255(6):828-30.

28. Hepper A. Confused patient, puzzled doctor. Journal of Hospital Medicine. 2012;7(Suppl 2):s145-s6.

29. Hoffman MA, Valderrama E, Fuchs A, Friedman M, Rai K. Leukemic Meningitis in B-Cell Prolymphocytic Leukemia A Clinical, Pathologic, and Ultrastructural Case Study and a Review of the Literature. Cancer. 1995;75:1100-3.

30. Imitola J, Pitt K, Peoples JL, et al. Multifocal CNS infiltration of chronic lymphocytic leukemia in the form of small-cell solid metastatic lesions. J Neurooncol. 2012;109(1):213-5.

31. Jhaveri D, Swayne A, Brown H, Airey C. A case of severe optic neuropathy caused by biopsy proven cll without peripheral transformation with excellent treatment response to rituximab, obinutuzumab (second generation anti-cd20 monoclonal antibody) and chlorambucil. Journal of Neurology, Neurosurgery & Psychiatry. 2017;88(5):e1.86-e1.

32. Kaiser U. Cerebral Involvement as the Initial Manifestation of Chronic Lymphocytic Leukaemia. Acta Haematologica. 2003;109(4):193-5.

33. Kakimoto T, Nakazato T, Hayashi R, et al. Bilateral Occipital Lobe Invasion in Chronic Lymphocytic Leukemia. Journal of Clinical Oncology. 2010;28(3):e30-e2.

34. Kalac M, Suvic-Krizanic V, Ostojic S, Kardum-Skelin I, Barsic B, Jaksica B. Central nervous system involvement of previously undiagnosed chronic lymphocytic leukemia in a patient with neuroborreliosis. Int J Hematol. 2007;85(4):323-5.

35. Khan K, Malik AI, Almarzouqi SJ, et al. Optic Neuropathy Due to Chronic Lymphocytic Leukemia Proven With Optic Nerve Sheath Biopsy. J Neuroophthalmol. 2016;36(1):61-6.

36. Kiewe P, Dallenbach FE, Fischer L, et al. Isolated B-Cell Lymphoproliferative Disorder at the Dura Mater with B-Cell Chronic Lymphocytic Leukemia Immunophenotype. Clinical Lymphoma and Myeloma. 2007;7(9):594-6.

37. Knop S, Herrlinger U, Ernemann U, Kanz L, Hebart H. Fludarabine may induce durable remission in patients with leptomeningeal involvement of chronic lymphocytic leukemia. Leuk Lymphoma. 2005;46(11):1593-8.

38. Korsager S, Laursen B, Mortensen TM. Dementia and central nervous system involvement in chronic lymphocytic leukaemia. Scandinavian Journal of Haematology. 1982;29(4):283-6.

39. Krawczyk-Kuliś M, Kopińska A, Dziaczkowska-Suszek J, Krycz-Krzemień S. Flow cytometry for diagnosis of a rare case of chronic lymphocytic leukaemia presenting in the central nervous system and effective treatment with liposomal cytarabine. The American Journal of Case Reports. 2011;12:145-9.

40. Krisht K. Combined chronic lymphocytic leukemia and prolactinoma: a rare occurrence in a patient presenting with pituitary apoplexy. J Neurosurg. 2013;119:924-8.

41. Kuwabara H, Kanamori H, Takasaki H, et al. Involvement of central nervous system in prolymphocytoid transformation of chronic lymphocytic leukemia. Leuk Lymphoma. 2003;44(7):1235-7.

42. Lange CP, Brouwer RE, Brooimans R, Vecht Ch J. Leptomeningeal disease in chronic lymphocytic leukemia. Clin Neurol Neurosurg. 2007;109(10):896-901.

43. Lannemyr O, Acosta S, Birgegård G, Hagberg H. Successful treatment of syndrome of inappropriate antidiuretic secretion (SIADH) in 2 patients with CNS involvement of chronic lymphocytic leukaemia. Eur J Haematol. 1997;58:207-8.

44. Liepman MK, Votaw ML. Meningeal Leukemia Complicating Chronic Lymphocytic Leukemia. Cancer. 1981;47:2482-4.

45. Lopez-Guillermo A, Cervantes F, Blade J, et al. Central nervous system involvement demonstrated by immunological study in prolymphocytic variant of chronic lymphocytic leukemia. Acta Haematologica. 1989;81(2):109-11.

46. Majumdar G. Chronic lymphocytic leukaemia with cerebral infiltration. Leuk Lymphoma. 1998;28(5-6):603-5.

47. Marmont AM. Leukemic meningitis in B-cell chronic lymphocytic leukemia: resolution following intrathecal methotrexate. Blood. 2000;96(2):776-7.

48. Matas A, Almendra R, Veiga A, Costa A, Gabriel JP, Da Silva MR. Extradural compressive myelopathy associated with B-cell chronic lymphocytic leukemia: A rare complication of a common malignancy. European Journal of Neurology. 2015;22(Suppl 1):530.

49. Matsuo T, Yamaoka A, Shiraga F, Matsuo N. Two types of initial ocular manifestations in intraocular central nervous system lymphoma. Retina, the journal of retinal and vitreous diseases. 1998;18(4):301-7.

50. Mehrpour G, Hosseinzade M. CNS involvement by chronic lymphocytic leukemia presented as intractable epilepsy: 'a case report'. Brain pathology. 2010;20(Suppl 1):87.

51. Miller K, Budke H, Orazi A. Leukemic meningitis complicating early stage chronic lymphocytic leukemia. Archives of Pathology and Laboratory Medicine. 1997;121(5):524-7.

52. Moazzam AA, Drappatz J, Kim RY, Kesari S. Chronic lymphocytic leukemia with central nervous system involvement: report of two cases with a comprehensive literature review. J Neurooncol. 2012;106(1):185-200.

53. Monteiro A, Gomes T, Fitas D, Nadais G. Small lymphocytic lymphoma associated with extensive myelopathy: Case report and review of the literature. J Neurol Sci. 2016;369:283-5.

54. Morrison C, Shah S, Flinn IW. Leptomeningeal Involvement in Chronic Lymphocytic Leukemia. Cancer Practice. 1998;6(4):223-8.

55. Mowatt L, Matthews T, Anderson I. Sustained Visual Recovery After Treatment With IntrathecalMethotrexate in a Case of Optic Neuropathy Caused by Chronic Lymphocytic Leukemia. J Neuro-Ophtalmol. 2005;25(2):113-5.

56. Nemoto K, Ohnishi Y, Tsukada T. Chronic lymphocytic leukemia showing pituitary tumor with massive leukemic cell infiltration, and special reference to clinicopathological findings of CLL. Acta Pathologica Japonica. 1978;28(5):797-805.

57. Patton WN, Carey MP, Fletcher MR, et al. Diffuse intracerebral involvement in B-cell chronic lymphocytic leukaemia. A case report. Clin lab Haemat. 1992;14:149-54.

58. Pérez Fernández I, Salgado Odóñez F, Rueda Dominguez A, Sevilla García I, Siles Rodriguez A. Infiltration of the Central Nervous System as a Presentation Form of Early Stage Chronic Lymphocyte Leukemia. American Journal of Hematology. 1998;58:339-42.

59. Pohar S, deMetz C, Poppema S, Hugh J. Chronic lymphocytic leukemia with CNS involvement. Journal of Neuro-Oncology. 1993;16:35-7.

60. Pohlen M, Kerkhoff A, Kessler T, Müller-Tidow C, Berdel WE, Koschmieder S. Leptomeningeal involvement of chronic lymphocytic leukemia: A report of two

patients successfully treated with combined chemotherapy and liposomal cytarabine (DepoCyte). Onkologie. 2010;33(suppl 6):242.

61. Poplawska-Szczyglowska L, Walewski J, Pienkowska-Grela B, Rymkiewicz G, Mioduszewska O. Chronic lymphocytic leukaemia presenting with central nervous system involvement. Medical Oncology. 1999;16:65-8.

62. Rassidakis G, Sepsa C, Kyrtsonis M-C, et al. Rare involvement of central nervous system (CNS) in typical chronic lymphocytic leukemia (CLL): report of a case and literature review. Virchows Arch. 2011;459(Suppl 1):S103-S4.

63. Remková A, Bezayová T, Vyskočil M. Brief report - B cell chronic lymphocytic leukemia with meningeal infiltration by T lymphocytes European Journal of Internal Medicine. 2003;14:49-52.

64. Rojas-Hernandez CM, Nemunaitis J, Marjon KD, Bustamante D, Zhang QY, Gillette JM. Chronic lymphocytic leukemia with clinical debut as neurological involvement: a rare phenomenon and the need for better predictive markers. BMC Hematol. 2017;17:3.

65. Rossi C, Brisou G, Baseggio L, et al. Central nervous system involvement in chronic lymphocytic leukemia: uncommon manifestation with undefined therapeutic management. Leuk Lymphoma. 2014;55(8):1939-41.

66. Ruiz P, Moezzi M, Chamizo W, Ganjei P, Whitcomb CC, Rey LC. Central nervous system expression of a monoclonal paraprotein in a chronic lymphocytic leukemia patient. Acta Haematologica. 1992;88(1):37-40.

67. Russwurm G, Heinsch M, Radkowski R, et al. Dasatinib induces complete remission in a patient with primary cerebral involvement of B-cell chronic lymphocytic leukemia failing chemotherapy. Blood. 2010;116(14):2617-8.

68. Rye AD, Stitson RNM, Dyer MJS. Pituitary infiltration in B-cell chronic lymphocytic leukaemia. British Journal of Haematology. 2001;115:718.

69. Schmidt-Hieber M, Thiel E, Keilholz U. Spinal paraparesis due to leukemic meningitis in early-stage chronic lymphocytic leukemia. Leuk Lymphoma. 2005;46(4):619-21.

70. Scully RE, Mark EJ, McNeely WF, McNeely BU. Case records of the massachusetts general hospital, Case 4 - 1993. The New England Journal of Medicine. 1993;328(4):266-75.

71. Singh AK, Thompson RPH. Leukaemic meningitis in chronic lymphocytic leukaemia. Acta Haematologica. 1986;75(2):113-5.

72. Smiljanic M, Todorovic Balint M, Antic D, et al. Chronic lymphocytic leukemia involvement of central nervous system: a single centre experience. Hematological Oncology. 2017;35 (Supplement 2):386.

73. Solal-Celigny P, Schuller E, Courouble Y, Gislon J, Elghozi D, Boivin P. Cerebromeningeal location of chronic lymphoid leukemia. Rapid immunochemical diagnosis and complete remission by intrathecal chemotherapy. Presse Med. 1983;12(37):2323-5.

74. Stagg MP, Gumbart CH. Chronic Lymphocytic Leukemic Meningitis as a Cause of the Syndrome of Inappropriate Secretion of Antidiuretic Hormone. Cancer. 1987;60:191-2.

75. Strati P, Uhm JH, Kaufmann TJ, et al. Prevalence and characteristics of central nervous system involvement by chronic lymphocytic leukemia. Haematologica. 2016;101(4):458-65.

76. Tam CS, Kimber T, Seymour JF. Ibrutinib monotherapy as effective treatment of central nervous system involvement by chronic lymphocytic leukaemia. Br J Haematol. 2017;176(5):829-31.

77. Thavapalan V, Kenyon L, Mizrahi M. Non-richter's transformation cll in the CNS: A case series. Neurology. 2014;82(10 suppl. 1).

78. Thiruvengadam R, Bernstein ZP. Central Nervous System involvement in Prolymphocytic transformation of Chronic Lymphocytic Leukemia. Acta Haematologica. 1992;87:163-4.

79. Turgut B, Pamuk GE, Turgut N, Demir M. Chronic lymphocytic leukemia with central nervous involvement in the form of localized mass responding to therapy with fludarabine. Yeni Symposium 2007. 2007;45(2):80-3.

80. Vora R, Khan M, Parshad S, Hollweg A, Rai K, Barrientos J. Neurological involvement in chronic lymphocytic leukemia: A report on 6 cases. Journal of Investigative Medicine. 2013;61(3):673-4.

81. Wang M-L, Shih L-Y, Dunn P, Kuo M-C. Meningeal involvement in B-cell chronic lymphocytic leukemia: Report of two cases. Journal of the Formosan Medical Association. 2000;99(10):775-8.

82. Wanquet A, Birsen R, Bonnet C, et al. Management of central nervous system involvement in chronic lymphocytic leukaemia: a retrospective cohort of 30 patients. Br J Haematol. 2017;176(1):37-49.

83. Wanquet A, Birsen R, Lemal R, Hunault M, Leblond V, Aurran-Schleinitz T. Ibrutinib responsive central nervous system involvement in chronic lymphocytic leukemia. Blood. 2016;127(19):2356-8.

84. Watanabe N, Takahashi T, Sugimoto N, et al. Excellent response of chemotherapy-resistant B-cell-type chronic lymphocytic leukemia with meningeal involvement to rituximab. Int J Clin Oncol. 2005;10(5):357-61.

85. Witton L, Menon S, Perera S. A rare case of CNS involvement with Chronic Lymphocytic Leukemia CLL. Neurology. 2017;88(16 Supplement 1).

86. Zaidi AS, Burton EC, Lotia M. Leptomeningeal disease as a manifestation of CLL. Journal of Clinical Oncology. 2012;30(15 suppl. 1).

87. Zawilska K. Central Nervous System Infiltration in a Patient with Chronic Lymphocytic Leukemia. Clinical Lymphoma, Myeloma & Leukemia Supplement. 2011(3.26):S215.

88. Zhou W, Niu X, Wang Z. Infiltration of central nervous system in chronic lymphocytic leukemia: Case report and review of literature. Chinese Journal of Clinical Oncology. 2013;40(22):1400-3.

89. Zhu J, Wu Z, Fan L, Li J. Chronic lymphocytic leukemia with central nervous system invasion: one case report and literature review. Zhonghua xue ye xue za zhi. 2014;35(7):592-5.
